# Overexpression of an Immune Checkpoint (CD155) in Breast Cancer Associated with Prognostic Significance and Exhausted Tumor-Infiltrating Lymphocytes: A Cohort Study

**DOI:** 10.1155/2020/3948928

**Published:** 2020-01-13

**Authors:** Yu-Chen Li, Quan Zhou, Qing-Kun Song, Rui-Bin Wang, Shuzhen Lyu, Xiudong Guan, Yan-Jie Zhao, Jiang-Ping Wu

**Affiliations:** ^1^Department of Breast Surgery, Beijing Shijitan Hospital, Capital Medical University, Beijing, China; ^2^Sid Faithfull Brain Cancer Research Laboratory, QIMR Berghofer Medical Research Institute, Queensland, Australia; ^3^Department of Pathology, Beijing Shijitan Hospital, Capital Medical University, Beijing, China; ^4^Department of Clinical Epidemiology and Evidence-based Medicine, Beijing Shijitan Hospital, Capital Medical University, Beijing, China; ^5^Division of Epidemiology, Beijing Key Laboratory of Cancer Therapeutic Vaccine, Beijing, China; ^6^Department of Emergency, Beijing Shijitan Hospital, Capital Medical University, Beijing, China; ^7^Department of Neurosurgery, Beijing Tiantan Hospital, Capital Medical University, Beijing, China

## Abstract

**Purpose:**

The immune checkpoint inhibitor is approved for breast cancer treatment, but the low expression of PD-L1 limits the immunotherapy. CD155 is another immune checkpoint protein in cancers and interacts with ligands to regulate immune microenvironment. This study is aimed at investigating the expression of CD155 and the association with prognosis and pathological features of breast cancer.

**Methods:**

126 patients were recruited this cohort study consecutively, and CD155 expression on tumor cells was detected by immunohistochemistry. The Kaplan-Meier survival curve and Cox hazard regression model were used to estimate the association.

**Results:**

38.1% patients had an overexpression of CD155, and the proportion of tumor cells with CD155 overexpression was 17%, 39%, 37%, and 62% among Luminal A, Luminal B, HER2-positive, and triple negative breast cancer cases, respectively (*p* < 0.05). Patients with CD155 overexpression had the Ki-67 index significantly higher than that of patients with low expression (42% vs. 26%). Though the number of tumor-infiltrating lymphocytes was higher among patients with CD155 overexpression (144/HPF vs. 95/HPF), the number of PD-1^+^ lymphocytes was significantly higher (52/HPF vs. 25/HPF, *p* < 0.05). Patients of CD155 overexpression had the disease-free and overall survival decreased by 13 months and 9 months, respectively (*p* < 0.05). CD155 overexpression was associated with an increased relapse (HR = 13.93, 95% CI 2.82, 68.91) and death risk for breast cancer patients (HR = 5.47, 1.42, 20.99).

**Conclusions:**

Overexpression of CD155 was correlated with more proliferative cancer cells and a dysfunctional immune microenvironment. CD155 overexpression introduced a worse relapse-free and overall survival and might be a potential immunotherapy target for breast cancer.

## 1. Introduction

In 2018, atezolizumab was approved to treat the triple negative breast cancer (TNBC) patients with PD-L1 expression [1]. However, the proportion of TNBC is less than 20% [2] and the expression rate of PD-L1 is less than 20% among BC patients [3–5]. The percentage of BC patients who are eligible to received immune checkpoint inhibitor is less than 5%. The immune checkpoint inhibitor targeting the PD-1/PD-L1 pathway is limited for immunotherapy among BC patients.

CD155 is another immune checkpoint protein, expressing on tumor cells and interacts with CD96, CD226, and T cell immunoreceptor with immunoglobulin and ITIM domains (TIGIT) on tumor-infiltrating lymphocytes to modulate the immune function in tumor immune microenvironment [6–8]. CD155, also known as the poliovirus receptor (PVR) or Nectin-like molecule 5 (Necl5), has been identified as an unfavourable prognosis marker and has an overexpression in a number of cancers, including glioblastoma multiforme [9], non-small-cell lung carcinoma [10], pancreatic cancer [11], melanoma [12], hepatocellular carcinoma [7], colorectal cancer [13], and sarcoma [14, 15]. CD155 is a cell adhesion molecule of the immunoglobulin-like superfamily and exerts cell-intrinsic activities that promote tumour growth and metastasis [16]. Expression of CD155 was seldom reported to be related with the inhibitory immune function in tumor microenvironment of BC. Here, we have investigated the expression of CD155 in BC tissues and the association with pathological characteristics, immune function of tumor microenvironment, and survival, in order to explore the immunotherapy potence of the CD155 pathway among BC patients.

## 2. Methods

All procedures performed in this study involving human participants were approved by the ethical committee of Beijing Shijitan Hospital, Capital Medical University, in accordance with the ethical standards of the 1964 Helsinki declaration and its later amendments. This study was under a retrospective study and the formal consent was waivered.

### 2.1. Patients

126 patients with invasive ductal BC were recruited into this cohort study from January 1, 2012 to December 31, 2013 consecutively. Patients were diagnosed with operable BC and received surgical treatment at the Department of Breast Surgical Centre of Beijing Shijitan Hospital, Capital Medical University. All the cases were diagnosed with primary invasive BC based on histological features, and tumours were graded according to the Nottingham modification of the Bloom–Richardson system by 2 pathologists.

The surgical specimen from all patients was fixed by 4% neutral formaldehyde and embedded for paraffin (FFPE) sectioning.

### 2.2. Immunohistochemistry (IHC)

Expression of CD155 and PD-1 was detected by IHC on FFPE tumours. Immunostaining was done after dewaxing and rehydrating slides. Monoclonal antibody against CD155 (rabbit anti-human, #81254) was purchased from Cell Signalling Technology and monoclonal antibody against PD-1 (mouse anti-human, #UMAB199), CD4 (rabbit anti-human, #EP204), CD8 (rabbit anti-human, #SP16), and Ki-67 (mouse anti-human, #MIB1) were purchased from Beijing Zhong Shan Golden Bridge Biotechnology Co. Ltd. EnVision™ FLEX Target Retrieval Solutions were used for antigen retrieval. Endogenous peroxidase was blocked with 3% H_2_O_2_ at room temperature for 15 min.

### 2.3. IHC Scoring

Two pathologists estimated tumor-infiltrating lymphocytes (TILs) locating in the areas within the borders of the invasive tumor, excluding the zones with crush artifacts, necrosis, regressive hyalinization, and biopsy site. All mononuclear cells (including lymphocytes and plasma cells) were scored, while polymorphonuclear leukocytes were excluded. If the scoring was inconsistent between the two pathologists, a third higher-level pathologist evaluated the IHC test. An average number of TILs were counted in 10 high-power fields (HPF, ×400) in IHC sections, selected randomly.

Positive CD155 expression was recorded as brown membrane in tumor cells. Weak/incomplete staining was recorded as +, weak/complete or strong/incomplete staining was recorded as ++, and strong/complete was recorded as +++. Weak/complete or strong/incomplete staining on cell membrane of tumor cells was defined as overexpression. Percentage of tumor cells overexpressing CD155 was to estimate the proportion of tumor cells expressing as ++ and +++ on the whole slide. Positive PD-1 expression was recorded as brown cytoplasm in lymphocytes. Positive CD4 and CD8 expression was recorded as red cytomembrane in lymphocytes and double staining of CD4/PD-1 and CD8/PD-1 showed red cytomembrane and brown cytoplasm of lymphocytes. Expression rate of PD-1 CD4 and CD8 was estimated by calculating the positive PD-1, CD4, and CD8 cells among 100 TILs. We counted PD-1-positive cells among 100 CD4^+^ or CD8^+^ lymphocytes and calculated the proportion. The cell counts of phenotypic TILs were calculated by the particular proportion multiplied by the number of TILs. Ki-67 expression was defined as brown nucleus in BC cells and Ki-67 index was measured as the proportion of Ki-67 expression among 1000 BC cells.

A breast surgeon from the Department of Breast Surgery conducted the follow-up procedure on cancer recurrence and mortality every six months. The follow-up data was obtained at clinic visit, hospital records, and telephone interview. Breast cancer recurrence was defined by biopsy, bone scanning, and CT/MRI. The all-cause death data was obtained from patients and caregivers. The loss of follow-up rate was 10.3%.

### 2.4. Statistical Analysis

All analyses were conducted with SPSS software (version 17.0). Age, histological grade, and TNM stage were analyzed with CD155 expression by the Spearman correlation test. Correlation of ER, PR, and HER2 status and CD155 expression was analyzed by the Mann–Whitney *U* test. The relationship between CD155 expression and molecular subtype was estimated under the Kruskal-Wallis test, and pairwise comparisons were conducted with Luminal A by Dunn's multiple comparison test. Difference of Ki-67 index, cell counts of TILs, percentage of PD-1^+^ TILs, and cell counts of PD-1^+^ TILs between CD155 expression status were estimated by the Mann–Whitney *U* test. The correlation between cell counts of CD4^+^, CD4^+^/PD-1^+^, CD8^+^ and CD8^+^/PD-1^+^ TILs, and CD155 expression was conducted by the Spearman correlation test. Kaplan-Meier survival curve was used to estimate the survival difference of patients classified by CD155 expression. Cox hazard regression model was used to calculate the hazard ratio (HR) and 95% confidence interval (95% CI) of CD155 status with age, histological grade, and TNM stage adjustment. All analyses were two sided and the significance level was 0.05.

## 3. Results

**Figure 1:**
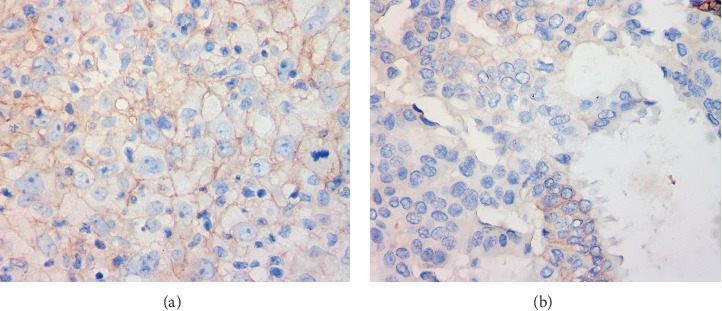
Classification of CD155 expression on BC tissues. (a) Overexpression of CD155. (b) Low expression of CD155.

A total of 126 cases diagnosed with invasive BC were evaluated with CD155 expression, 38.1% (48) of the cases were stained as overexpression (Figure 1(a)), whereas 61.9% (78) were stained as low expression (Figure 1(b)). Intratumoral and stromal immune cells which were found highly involved in BC tumour environment were negative for CD155 expression.

The diagnosis age of BC patients had not any association with CD155 expression (Table 1). The percentage of tumor cells with CD155 overexpression was 5% among patients at grade I and increased to 42% among patients at grade III (*p* < 0.05, Table 1). ER expression status had a significant relationship with CD155 expression status (Table 1). The percentage of tumor cells with CD155 overexpression was 56% and 21% among BC patients with negative and positive ER, respectively (*p* < 0.001). CD155 expression status did not show any difference between the status of PR, HER2, or TNM stage (Table 1).

**Figure 2:**
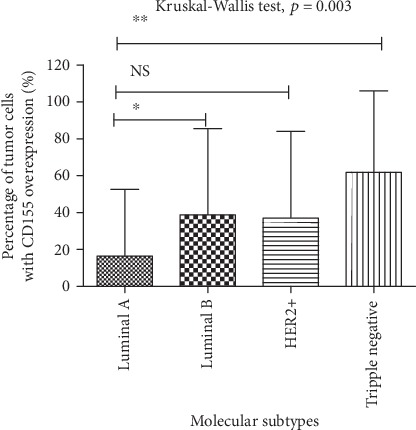
Distribution of percentage of tumor cells overexpressing CD155 between molecular subtypes of breast cancer.

The percentage of tumor cells overexpressing CD155 was 17%, 39%, 37%, and 62% among Luminal A, Luminal B, HER2-positive, and TNBC cases, respectively (*p* < 0.05, Figure 2). The percentage was higher in Luminal B and TNBC cases than that in Luminal A patients (Figure 2).

**Figure 3:**
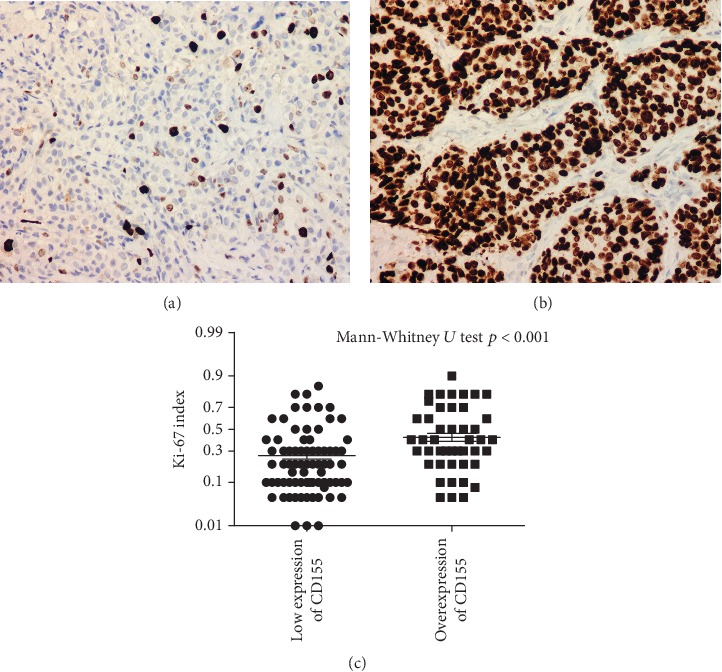
Relationship between Ki-67 index and CD155 status. (a) Ki-67 expression among patients with low expression of CD155. (b) Ki-67 expression among patients with overexpression of CD155. (c) The difference of Ki-67 expression between patients with low expression and overexpression of CD155.

**Figure 4:**
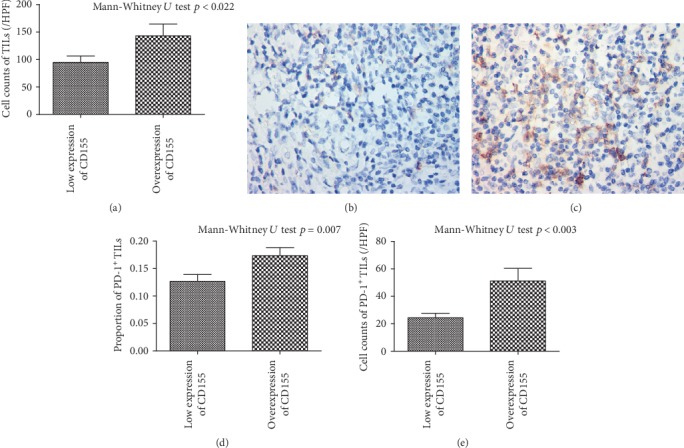
Relationship between expression of CD155, cell counts of TILs, and PD-1 expression. (a) Cell count of TILs. (b) PD-1 status among patients with low expression of CD155. (c) PD-1 status among patients with overexpression of CD155. (d) Percentage of PD-1^+^ TILs. (e) Cell count of PD-1^+^ TILs.

Among patients with low expression of CD155, Ki-67 index was 26% (Figure 3(a)), significantly lower than patients with CD155 overexpression (42%, Figures 3(b) and 3(c)). Cell count of TILs was 144/HPF among patients with CD155 overexpression, in comparison of 95/HPF among patients with low expression of CD155 (*p* < 0.05, Figure 4(a)). However, the percentage of PD-1^+^ TILs was significantly higher (17% vs. 13%) in patients with CD155 overexpression than that of patients with low expression of CD155 (*p* < 0.01, Figures 4(b)–4(d)) and the cell count of PD-1^+^ TILs was 52/HPF and 25/HPF among patients with overexpression and low expression of CD155, respectively (*p* < 0.01, Figures 4(b), 4(c), and 4(e)). The percentage of tumor cells overexpressing CD155 had a significant correlation with cell counts of CD4^+^, CD4^+^/PD-1^+^, CD8^+^, and CD8^+^/PD-1^+^ TILs (Table 2).

**Figure 5:**
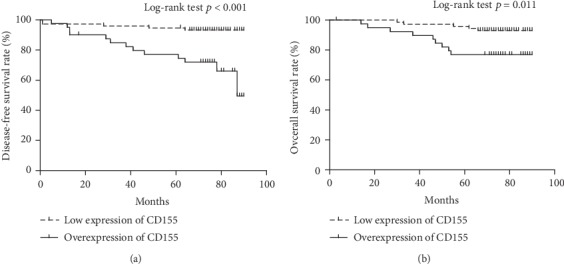
Effects of CD155 expression on disease-free and overall survival. (a) Disease-free survival. (b) Overall survival.

The median follow-up time was 75 months, and the rate of loss of follow-up was 7.1% and 10.3% for relapse and overall survival, respectively (Table 2). The mean DFS length was 86 months among patients with low expression of CD155, significantly longer than patients (73 months) with CD155 overexpression (Figure 5(a)). The mean OS length was 87 months among patients with low expression of CD155, significantly longer than patients (78 months) with CD155 overexpression (Figure 5(b)).

The DFS and OS rate was 88.5% and 87.2% among patients with low expression of CD155; however, the survival rate reduced to 56.3% and 62.5% among patients with overexpression of CD155 (Table 3). The study power was 97.7% and 87.3% for DFS and OS survival rate. In Cox hazard regression analysis, the overexpression of CD155 was associated with a 5.41-fold high risk of relapse (95% CI 1.93, 15.20) and a 3.74-fold high risk of death (95% CI 1.25, 11.16); under further adjustment, the HR of relapse increased to 13.93 (2.82, 68.91) and the HR of death increased to 5.47 (95% CI 1.42, 20.99) (Table 3).

## 4. Discussion

CD155 is another immune checkpoint protein, characterized as a type I transmembrane glycoprotein belonging to the immunoglobulin superfamily and expressed in many cancer cells. In this study, CD155 had an overexpression in BC and correlated with higher PD-1, Ki67 expression, and poorer survival.

CD155 expression was related with vascular endothelial growth factor (VEGF) level and played a role in angiogenesis of pancreatic cancer cells [11]. The Ras-Raf-MEK-ERK signalling [17], sonic hedgehog signalling [18], and Toll-like receptor signalling pathways [19] were reported to affect the expression of CD155. Moreover, DNA damage has been shown to induce CD155 expression. Several chemotherapeutic reagents against BC, such as adriamycin, were shown to induce CD155 expression [20]. The domain structure of CD155 is similar to nectin and implicated in organizing cell adhesion junctions and cell polarization [21]. In particular, its overexpression enhances cancer cell migration and proliferation [17].

CD155 is the molecule expressing during embryonic period, which barely expresses in normal tissues, but reexpresses in malignant tissues [9, 11–13, 22]. Moreover, soluble isoforms of CD155 have been found to be highly expressed in the sera of patients with lung, gastrointestinal, breast, and gynaecologic cancers than that of in healthy volunteers [23]. Furthermore, the expression level was significant higher in patients with advanced-stage cancers (stages III and IV) than those with early-stage cancers (stages I and II) [23]. However, the expression level decreased after surgical resection, indicating it was a potential biomarker for cancer progression and prognosis.

CD155 was reported to overexpress in hepatocellular carcinoma, and lower density was correlated with a better DFS and OS [7]. Among patients with pancreatic cancer, low expression of CD155 was associated with a median DFS of 22.2 months and high expression was associated with a median DFS of 14.0 months [11]. In addition, the correlation with a worse survival indicated CD155 was a prognostic biomarker for lung cancer, sarcoma, melanoma, and GBM. CD155 expression promoted the tumor growth and metastasis. Patients of osteosarcoma with lung metastasis had higher expression of CD155 than those with primary osteosarcoma, and CD155 expression was correlated with the tumor size [15]. The blockade of CD155 molecules even reduced the number of metastatic nodules in the lung [15]. Downregulation of CD155 in gastric cancer cells inhibited tumor progression and improved the survival of treated mice [6]. Intratumoral treatment of the recombinant nonpathogenic polio-rhinovirus chimera to patients with recurrent glioblastoma prolonged the survival length, comparing with the historical controls [24]. The prognostic effect of CD155 expression on BC was confirmed in another two publications [25, 26]. The two articles presented a significant association between CD155 expression with NK [25] and cytotoxic and macrophage-TILs [26]; however, we not only observed a significant relationship with TILs but also a significant relationship with PD-1^+^ TILs (dysfunctional TILs). Therefore, we concluded the exhausted TILs, but not the functional TILs related with CD155. The positive relationship with the dysfunctional TILs indicates an immune suppressive role of CD155 in the tumor microenvironment and provides a potential of immunotherapy for breast cancer. Furthermore, we analysed the exhausted helper and cytotoxic TILs and both had significant association with CD155 expression. These findings indicated that the exhausted effector TILs not the functional effector TILs were related with CD155 expression.

The endogenous function of CD155 in cancer is not well characterized. CD155 has been shown to play a key role in cancer migration, invasion, and metastasis [27–29]. Several proteins have been found to interact with CD155 during these biological processes. CD155 promoted tumor cell migration by colocalizing with *α*v-integrin, leading to assembly of focal adhesion complexes that stabilize cellular interaction with its substrate through intracellular signalling and rearrangement of the actin cytoskeleton [27]. In glioma animal models, overexpression of CD155 was involved in enhanced cell dispersal, reduced cell spreading, and focal adhesion density [29]. Moreover, expression of CD155 increased Src/focal adhesion kinase signalling and enhanced the adhesion-induced activation of paxillin and p130Cas in cells adhering to vitronectin [29]. In contrast, depletion of endogenous CD155 enhanced focal adhesion, induced cell spreading, and finally inhibited migration [27]. CD155 interacts with its ligands on immune cells and regulates immune function. TIGIT, CD96, and CD226 are common ligands of CD155; the TIGIT/CD96-CD155 pathway delivers an inhibitory signal to immune cells; however, CD226 delivers an activated signal [30]. The balance of the three ligands played an important role of immune homeostasis in tumor microenvironment [31]. It has recently been reported that the interaction of CD155 with TIGIT on T cell and natural killer (NK) cells induced an inhibition on cell proliferation, cytotoxicity [32, 33], and immune functional exhaustion [6]. The interaction of CD155 with TIGIT or CD96 on T cell and NK cells exhausted the immune function and reduced production of interferon *γ* (IFN*γ*), tumor necrosis factor *α* (TNF*α*), and other cytokines [6, 7, 34, 35]. The blockade of CD155-TIGIT/CD96 signalling could restore the immune and cytotoxic function of NK cells [6, 7, 34, 35]. CD226 expresses on T cell and NK cells, is bound to CD155, and enhances cytotoxic function [7, 8]. The interaction increased NK-mediated suppression of melanoma metastasis [36]. CD155 mediated NK cell cytotoxicity through the AKT–FOXO1 pathway [37]. The immune regulatory role of CD155 might depend on the circumstances in the tumor microenvironment. The balance between activating and inhibitory signals maintains the normal function of immune cells, and the imbalance in the tumour microenvironment contributes to immune escape of tumor cells [38]. The immune checkpoint pathway of CD155-CD96/CD226/TIGIT is a potential immunotherapy target for BC.

CD155 played a vital role in increasing cell proliferation in ras-mutated cancer cells, by upregulating cyclin D2, activating of ERK signal, downregulating p27, and shortening the G_0_/G_1_ phase of the cell cycle [39]. On the other hand, CD155 downregulation inhibited proliferation and induced cell cycle arrest at G_2_/M phase [11]. Ki-67 is a proliferative cell nuclear antigen and presents expression in all mitotic phases of cells except G_0_ and early G_1_ phase [40]. The peak expression appears in M period [40]. High Ki-67 index indicated a severe malignant degree and proliferation activity of BC cells [41]. In our study, CD155 expression had a positive correlation with Ki-67 expression, because CD155 increased cancer cell proliferation and downregulation of CD155 reduced proliferation in BC and pancreatic cancer cells [11, 20].

In this study, CD155 expression was positively correlated with the number of TILs, not as the correlation in pancreatic cancers [11]. CD155 expression was related with an impaired immune function and we observed a positive association between CD155 and PD-1 expressions on TILs. CD155 has been implicated in a variety of cancers, but its biological role in BC development and progression is still unclear. CD155 knockdown induced BC cells apoptosis both in vitro and xenograft models [20]. The immunotherapy targeting the CD155-CD96, CD226, and TIGIT pathways is significant for BC patients.

The unclear expression of TIGIT, CD96, and CD226 on NK cells was the main limitation in this study. The related signalling pathway was not detected in the tissues. The limited sample size for BC molecular subtypes was another limitation.

## 5. Conclusion

CD155 had an overexpression in BC and associated with more proliferative cancer cells, a severer exhausted immune microenvironment, and higher risk of relapse and death. The immune checkpoint protein, CD155, is a potential immunotherapeutic target for BC.

## Figures and Tables

**Table 1:** Association between CD155 expression and clinical pathological features.

	*N*	Percentage of tumor cells with CD155 overexpression	*p*
Age, correlation coefficient^†^	126	-0.026	0.774
Histological grade^†^			
I	13	5% (17%)	0.049
II	79	32% (45%)	
III	26	42% (48%)	
ER status^‡^			
Negative	31	56% (47%)	<0.001
Positive	90	21% (39%)	
PR status^‡^			
Negative	41	35% (46%)	0.307
Positive	80	27% (43%)	
HER2 overexpression^‡^			
Negative	63	26% (42%)	0.223
Positive	21	40% (47%)	
TNM stage^†^			
I	30	23% (41%)	0.58
II	67	36% (46%)	
III	18	21% (38%)	
IV	6	50% (55%)	

^†^Spearman correlation test. ^‡^Mann–Whitney *U* test.

**Table 2:** Association between CD155 expression and clinical pathological features.

	*N*	Percentage of tumor cells with CD155 overexpression	*p*
Cell counts of CD4^+^ TILs, correlation coefficient^†^	126	0.226	0.011
Cell counts of CD4^+^/PD1^+^ TILs, correlation coefficient^†^	126	0.341	<0.001
Cell counts of CD8^+^ TILs, correlation coefficient^†^	126	0.213	0.017
Cell counts of CD8^+^/PD1^+^ TILs, correlation coefficient^†^	126	0.201	0.024

**Table 3:** Cox hazard regression on association between CD155 expression and prognosis.

	CD155 expression		HR_crude_	95% CI	HR^†^	95% CI	HR^‡^	95% CI
	Low expression (*n* = 78)	Overexpression (*n* = 48)						
Disease-free survival, *n* (%)								
Relapse	6 (7.7)	15 (31.3)	5.41	1.93, 15.20	5.68	2.01, 16.02	13.93	2.82, 68.91
Survival	69 (88.5)	27 (56.3)						
Overall survival, *n* (%)								
Death	6 (7.7)	9 (18.8)	3.74	1.25, 11.16	4.51	1.50, 13.63	5.47	1.42, 20.99
Survival	68 (87.2)	30 (62.5)						

^†^Further adjusting age. ^‡^Further adjusting age, histological grade, and molecular subtype.

## Data Availability

The data used to support the findings of this study are available from the corresponding authors upon request.
